# 

**Published:** 1915-11-13

**Authors:** 


### CONTENTS.

| | PAGE |
|---|---|
| **Junior Students and Recruiting** ... ... ... | 133 |
| **Women Doctors After the War** ... ... ... | 134 |
| **Hospital and Institutional News** ... ... ... | 135 |
| St. George's Hospital as Pioneer—Worthy of Universal Adoption—British Workmen or Mental Dements?—Civil Patients in War Time—A New Yorkshire Sanatorium—The Value of the Private Staff—No Driving Force at Worcester Infirmary—Beaufort War Hospital and the Bristol Guardians—An Age Limit at Radcliffe Infirmary—War Funds at Liverpool—Another British Hospital in the Argonne—The State Child—A Remarkable Hospital Magazine—Military Convalescents at Blackpool—Bath's Proposed War Hospital—Countess Temple's Protest—Medical Arrangements in Mesopotamia—The New Hospital in Oakdale Model Village—Hospital Wood-Carving—A Hospital Diamond Jubilee—Hospital Physician Changes His Name—A New Subject for Medical Students—Sanatorium Lady Superintendents—The Norfolk Supply Depots—The Doctor's Wife as "Locum"—School Closure Again—Portuguese Aid for British Wounded—The Cinema Ambulance Fund—This Week's Drug Market. | |
| **A Medical Causerie** ... ... ... ... ... | 141 |
| **Round the World** ... ... ... ... ... ... | 142 |
| Fellow-Workers and the Roll of Honour. | |
| **Patriotism in Medicine** ... ... ... ... | 143 |
| The Medical Schools of British Hospitals: St. Bartholomew's Medical School. | |
| **Legal Notes for Pharmacists** ... ... ... | 144 |
| **Education in the Transvaal** ... ... ... | 145 |
| Mr. C. Louis Leipoldt's Report as Medical Inspector. | |
| **Personalia** ... ... ... ... ... ... | 146 |
| **The Heating of Hospitals** ... ... ... ... | 147 |
| V.—The Royal Infirmary, Glasgow. | |
| **Buildings Contemplated** ... ... ... ... | 148 |
| **Parliament and the Profession** ... ... ... | 149 |
| **Passing Events and Topics** ... ... ... ... | 149 |
| **The London County Council** ... ... ... | 150 |
| **The Editor's Letter-Box** ... ... ... ... | 151 |
| About Rheuamtism—The Irish Matrons' Association and the Martyr Nurse—Chelsea Hospital for Women—The Army and Territorial Nurses' Cape—King George's Bed—An Extraordinary Case at Bradford. | |
| **The Institutional Worker** ... ... (Suppl.) | 1 |
| Work for Consumptives in Sanatoria:—Answer to September Question. | |
| Question for November ... ... (Suppl.) | 2 |
| Enquiries and Answers. | |
| Questions Invited ... ... ... (Suppl.) | 2 |

